# The Characteristics and Social Functioning of Pathological Social Withdrawal, “Hikikomori,” in a Secondary Care Setting: a One-Year Cohort Study

**DOI:** 10.1186/s12888-020-02660-7

**Published:** 2020-07-06

**Authors:** Hissei Imai, Toko Takamatsu, Hideaki Mitsuya, Hajime Yoshizawa, Hidehiko Mitsuya, Toshi A. Furukawa

**Affiliations:** 1 Health Promotion and Human Behavior, Kyoto University Graduate School of Medicine / School of Public Health, Yoshida Konoe-cho, Sakyo-ku, Kyoto, 606-8501 Japan; 2 Ohashi Psychiatric Clinic, Kyoto, Japan; 3 Mitsuya Psychiatric Clinic, Kyoto, Japan

**Keywords:** anxiety, apathy, culture-bound syndrome, anomie, hikikomori, social withdrawal, social dysfunction

## Abstract

**Background:**

Pathological social withdrawal, named “Hikikomori,” is a Japanese culture-bound syndrome and a serious social problem in Japan. The number of Hikikomori cases in Japan was estimated at about 563,000 in 2016 according to governmental surveys. However, no studies have reported how many people with Hikikomori have access to community-based psychiatry clinics, and how different they are from non-Hikikomori patients regarding their baseline characteristics and outcomes. The aim of the present study is to evaluate the baseline characteristics, clinical attendance, and social functioning of community psychiatric clinic patients treated for social withdrawal at one-year follow-up.

**Method:**

Participants (*n* = 304) were all patients (aged under 65) of a psychiatric clinic in a one-year period. Baseline patient characteristics were compared among “current” Hikikomori patients, “past” Hikikomori,” and “other” patients. Logistic regression analysis of clinic attendance status and social functioning at one-year follow-up was used to assess patient outcomes. Independent variables were age, gender, Hikikomori status, and support from clinical staff.

**Results:**

Numbers of “current”, “past” Hikikomori, and “other” patients were 60 (19.7%), 81 (26.6%), and 163 (53.6%), respectively. The percentage of “current” Hikikomori who attended in person (56.7%) was significantly smaller than for “past” (92.6%) and “other” (92.6) (*p* < .001). The age distribution of “current” Hikikomori patients was bimodal, peaking at 20 and 40–45 years. The “current” state predicted significantly fewer regular visits (OR = 0.43; 95% CI = 0.22–0.83; *p* = .012); support from psychiatric social workers increased visits (OR = 2.35; 95% CI = 1.14–4.86; *p* = .021). Among the “current” Hikikomori patients, first visit attendance in person predicted regular attendance; no factor consistently predicted working/schooling status.

**Conclusion:**

A sizable percentage of community clinic patients experienced Hikikomori. The “current” Hikikomori state corresponded with low clinic attendance and social function; “support from clinical staff” may increase visit regularity; no factors consistently improved social functioning. Further multi-site study is warranted to examine the generalizability of the findings from the current single-center study.

## Background

Pathological social withdrawal, named *Hikikomori*, was thought to be a Japanese culture-bound syndrome [21]. Although, it is now a serious social problem in other countries as well as in Japan [9]. *Hiki* means withdrawal and *Komori*, relates to *Komoru*, which denotes secluding or confining to a certain place. In 2010, the Japanese Ministry of Health, Labor, and Welfare defined Hikikomori as a condition that causes a person to withdraw into his/her home for six months or more, during which time they do not go to school, work, or participate in socializing. Originally, patients with Hikikomori were defined as having no psychiatric disorder. However, in the time that has passed since this condition was first recognized, many people with Hikikomori have been retrospectively identified as a having psychiatric disorder. Therefore, the Japanese Ministry of Health, Labor, and Welfare now describes it as a condition caused by many factors including psychiatric disorders. The narrow definition regarding people affected by the condition is, those who do not leave their rooms or homes, or who can go out in their neighborhoods but usually stay home. The broader definition is, those who go out for their hobbies but usually stay home.

There is a long history of Hikikomori in Japan. The problem known as “school refusal” drew the attention of educators and psychiatrists in Japan around the 1950s [7]. What is known presently as a Hikikomori-like state was already being reported at that time, but the word was not yet used. The psychiatrist Yomishi Kasahara proposed the concept of “retreat neurosis” in 1976, referring to a refusal of and withdrawal from social participation [8]. People with this disorder can participate from the sidelines. However, they withdraw from expected social roles and present with apathy, indifference, and depression. Tamaki Saito clearly defined the state of Hikikomori in 1990, attracting social interest, and the term was adopted as the clinical definition of the condition [20].

Many researchers have proposed ideas about the etiology of Hikikomori, but their ideas have largely been based on low-quality evidence, which consists of subjective opinions or from cross-sectional studies. For example, regarding the role of society in the manifestation of Hikikomori, some authors suggest that the high economic growth of the post-war period added to the emphasis in the education system on passing examinations to raise one’s social status [22]. However, this focus denied the diversity of life and society and excluded children who dropped out, veered away from his fixed path. The emphasis on exams and status essentially left such children behind and may have impelled them to be Hikikomori [22]. On the other hand, a maternal society marked by parents being overly protective may also lead to children being overly dependent. This prevents children from being independent and enables them to avoid society [15]. Others argue that young people’s inability to endure reality is worsened by non-working people having the same influence and right to speak out as people who work, encouraging them to claim their rights without any relationship to the real world [19]. Additionally, others refer to the characteristics of individualism as narcissistic, such that patients with Hikikomori retain a sense of omnipotence by avoiding reality [10]. It may also be that an unstable culture is caused by the dissociation between Japanese unconscious orientation to relationships and conscious orientation to individuality [11]. Alternatively, some cross-sectional studies have investigated the relationship of a patient’s Hikikomori with their father’s level of education, maternal psychiatric disorders [12], the patient’s educational level, income, general health, internet addiction symptoms [25], their experience of feeling “buried”, and weak family ties [14].

The number of Hikikomori (broad definition) cases in Japan was estimated at about 696,000 in 2010 and 563,000 in 2016 according to governmental surveys [2, 3]. Other studies indicated that the lifetime prevalence is 1.2% in Japan [12]. Although the majority of the population of Hikikomori used to be in their twenties, they are getting older, which is an economic and social problem in Japan [5]. The number of Hikikomori (broad definition) cases in people between the ages of 40 and 64 was estimated to about 613,000 in Japan in 2018 [4]. Hikikomori is not limited to Japan, it has been identified in Hong-Kong, Spain, France, India, Korea, and the U.S. [9, 14, 16, 25]. An investigation conducted in Hong-Kong showed that the point prevalence of Hikikomori was 1.7% [25]. A study conducted in Spain reported that 15.4% (200 out of 1297) of patients attended to the Crisis Resolution Home Treatment were Hikikomori case [16].

The biggest challenge is to improve social function of patients with Hikikomori. Community psychiatric clinics may play a major role in achieving this aim. However, it is unclear how different the outcomes would be for these patients compared to non-Hikikomori patients after clinic attendance, and, foremost, how different their status of clinic attendance would be relative to non-Hikikomori patients. Additionally, no studies have reported how many patients with Hikikomori are currently being treated in community-based psychiatry clinics. One key barrier to attending clinics may be anxiety. The results of a prior study imply that some children who first exhibit school refusal later shift towards more complete isolation [20] and that this avoidance of schools and clinics may both be associated with anxiety. To clarify these points, the aim of the present study is to evaluate the percentage of “current” and “past” Hikikomori patients in a community private clinic in a year and compare the background and the outcomes between Hikikomori and non-Hikikomori patients at one-year follow-up.

## Methods

### Subjects

We included new-visit patients aged below 65 in the Mitsuya psychiatric clinic in the year from June 1, 2017–May 31, 2018. We also included the data from family members in cases where they had visited on behalf of patients. The study protocol was reviewed by the Kyoto University ethical review board (R0855-1). As all the data used in the present study is from clinical records and interview sheet at the first visit, which had been collected as the daily practice at the clinic, the ethics committee approved a waiver for consent to participate for the study. Patients were informed of the study via the clinic’s website and the data of those who wish to be opted out were not included in the study. However, we included all new-visit patients under age 65, as none of them wished to opt-out from including their data in the study.

### Study location

The Mitsuya psychiatric clinic is a secondary care clinic in Neyagawa-city, Osaka prefecture, Japan. The city’s population as of June 1, 2017 was 236,483. The percentages of the population who were 14-years old or younger, between 15 and 64, and 65 and over were 12%, 59%, and 29%, respectively. Six doctors (four full-time and two part-time), nine nurses, nine psychiatric social workers, seven occupational therapists, five psychotherapists, and eight clerks work at the Mitsuya psychiatric clinic. The clinic has daycare floors and offers home visits. The clinic is a general psychiatric practice. Two psychiatrists see patients in the outpatient unit for six hours per day from Monday to Saturday. Additionally, some psychiatrists make home visits to patients who cannot come to the clinic. One psychiatrist is prepared to see patients with Hikikomori for two and a half hours on Wednesdays and Thursdays. However, sometimes there are no Hikikomori consultations scheduled. In this event, the doctor sees general patients during those designated times.

### Definition of Hikikomori

In determining eligibility for the study, we used the narrow definition of Hikikomori provided by the Japanese Ministry of Health, Labor and Welfare. It defines Hikikomori as those who do not go to a workplace or school, have no social interactions, stay in their homes for more than 6 months, and don’t leave their rooms/homes, or may go out into their neighborhoods but usually stay home.

Patients answered three questions asking if in the preceding six months they had, (a) gone to school or a workplace, (b) had any social interaction other than with family members and people at shops or hospitals, and (c) went out of the home more than rarely. The person was considered as “current Hikikomori” if all answers were no. Additionally, we asked the patients if there had been any earlier periods in which they would also answer no to all three questions. We determined that the person had experienced “past Hikikomori” if their answer was yes. The items equate to the definition of Hikikomori, which assures content validity. The Cronbach’s alpha based on the three items was 0.78, indicating acceptable reliability of the items.

### Variables

#### The Overall Anxiety Severity and Impairment Scale (OASIS)

The OASIS, a scale for assessing overall anxiety [18], consists of 5 items (each scored 0–5; higher scores = more severe symptoms). It addresses the frequency and severity of anxiety, avoidance, interference of ability at work, school, home, social life, and relationships in the past week. It has excellent test-retest reliability, convergent, and divergent validity. The Japanese version of OASIS has also been validated [6].

#### Other variables

Other variables included the basic demographics, experience of school refusal, the number of psychiatric clinics or hospitals each patient had visited to see a doctor, and their supporters during the follow-up period. School refusal was defined as the individual refusing to attend school for ≥ 30 days per year. Patients were determined to have experienced school refusal if they answered yes to the following question, “Have you experienced school refusal (total days of absence from school ≥ 30 days per year)?” Non-physician supporters were listed as home visiting nurse, psychiatric social worker (PSW), licensed psychotherapist, and occupational/physical therapist (OT/PT) during the follow-up period.

### Outcome variables

#### Attendance to the clinic

Attendance to the clinic was divided to seven categories: finalized consultation (patients recover and end consultation with doctors), regular visits (at least one visit per month three months before or after one year from the first visit), irregular visits (at least one visit three months before or after one year from the first visit), home visits by a doctor without patient visiting the clinic, continuous consultation by a person on behalf of patients (at least one consultation three months before or after one year from the first visit), transference to another clinic or hospital, discontinuation, or unknown.

#### Social functioning

Social functioning was divided to 6 categories: working/(includes work with support and part-time jobs)/going to school/homemaker, regular visit to day/night care without working; going out freely without regular visit to day/night care, going out in restricted time and place, indoor activity without going out, activity restricted to their room.

Day/night care offers rehabilitation for social participation. Primary activities include drawing pictures, playing music, participating in sports, cooking, and learning social skills. Some patients only come to the day/night room but do nothing. While there, others join various activities. Engaging in indoor activities without going out means that patients do not leave home, but they can do some activities inside the home. Usually, they meet with family members. Activity restricted to their rooms means that patients leave their rooms except to use the toilet or bathe. They rarely or never meet other family members. Meals are usually prepared by a family member outside patients’ rooms and patients take the meals when family member leave the room.

Data on diagnosis, clinic attendance, social functioning, and supports were collected from hospital records, and other data were collected from medical interview sheets completed by patients or their families at their first visit.

### Statistical analysis

We included all complete data and calculated the percentage of “current-” or “past- Hikikomori” patients among the new patients in a one-year period. First, we calculated the percentage of “current Hikikomori” patients. Second, we calculated “past” patients and “others” among the samples of the “without current Hikikomori” patients. The “presence in person/not in person” was also calculated for each category.

Variables were compared at baseline and at follow-up between Hikikomori and non-Hikikomori patients. We compared each patient’s gender, age, the number of clinic visits in the past year, experience of school refusal, state of anxiety, and diagnosis. However, state of anxiety and diagnosis were excluded from the analysis in case a surrogate person visited the clinic. Missing values were excluded. One-way ANOVA was used for parametric variables and the Bonferroni test was used as a post hoc comparison when there was a significant difference. The Kruskal-Wallis test and its adjusted version were used for non-parametric variables and post hoc comparison. The Chi-square test and the Ryan test were used for the comparison of percentages.

The primary purpose of analysis for the follow-up period was to clarify the influence of Hikikomori status on outcomes. Logistic regression was used to see the influence of variables on outcomes. Dependent variables were related to clinical visit (finalized consultation/regular visit or not, finalized consultation/regular visit/irregular visit/visit home by a doctor or not) and social function (work/school or not, work/school/daycare/night care or not). Independent variables were age, gender, Hikikomori status and support from a: nurse, PSW, OT/PT, and psychologist. Univariable logistic regression analysis was used for each independent variable and the multivariable logistic regression model for including all variables. The univariable logistic regression for “social function” was adjusted for the patient’s baseline social function.

All reported *p* values are two-tailed; *p* < .05 was the threshold for statistical significance. SPSS version 20 and R were used for the statistical analysis.

## Results

Among the 350 first-visit patients, 309 patients were under 65 years old. In all, 304 patients were included in the study analysis excluding 5 patients who did not answer the question identifying Hikikomori status. The number of “current” Hikikomori patients was 60 (19.7%). Excluding these patients, 81(26.6%) had experienced past Hikikomori. The percentage of patients who presented in person at the first visit was significantly smaller (*p* < .001) in current Hikikomori patients (*n* = 34, 56.7%) compared to other patients (past Hikikomori patients, *n* = 75, 92.6%; others, *n* = 151, 92.6%).

**Fig. 1:**
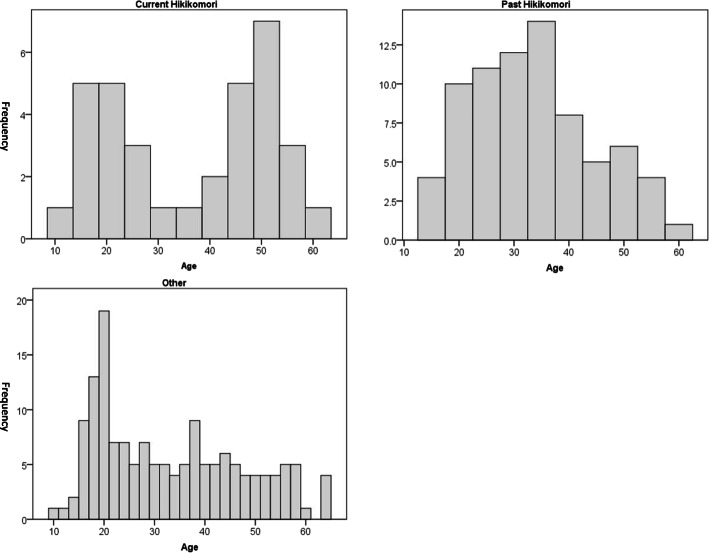
Age distribution of “current”, “past” Hikikomori, and other patients

Table 1 shows the comparison of basic demographics among all Hikikomori patients and “others.” The distribution of age was unusual, with age of “current” Hikikomori patients being bimodal, the most frequent ages being 20 and, 40-45, whereas those of past Hikikomori patients and others were left-skewed and the peak frequency was at 22.5 and 20, respectively (Fig. 1). The percentages of patients with past school refusal were significantly higher in “current” (*n* = 31, 59.6%) and “past” Hikikomori patients (*n*=35, 50.0%) than in “others” (*n* = 28, 17.8%, *p* < .001). The number of clinic visits was significantly larger in past Hikikomori patients (median = 1.0, interquartile range (IQR = 0-3.0) than in others (median = 1.0, IQR = 0-1.0, *p* = .002). The Bonferroni correction for multiple comparison setting the significance threshold as *p* < 0.0125 resulted in no significant difference in the number of clinic visits among groups.

**Table 1:** *Comparison of basic demographics among Hikikomori patients and others in a community psychiatric clinic.*

	Current Hikikomori		Past Hikikomori		Others		p
	n		n		n		
Age, median (IQR)	60	34.0 (21.3-46.8)	81	32.0 (23.0-40.5)	163	29.0 (20.0-43.0)	.31
Female, n(%)	60	27 (45.0)	81	43 (53.1)	163	86 (52.8)	.55
School refusal, n(%)	52	31 (59.6)^*^	70	35 (50.0)^#^	157	28 (17.8)^*,#^	< .001
Number of Clinic visits, median (IQR)	52	1.0 (0-2.0)	74	1.0 (0-3.0)^+^	133	1.0 (0-1.0)^+^	.002

*Notes.* p: ANOVA for parametric outcomes and Kruskal-Wallis test for non-parametric outcomes; *,#post hoc Ryan test, *p* < .001; +post hoc adjusted Kruskal-Wallis test *p* = .002. Symbols are matched to indicate where, for the indicated two groups, the difference between those groups were significant. *Abbreviations*: *IQR* Interquartile range, *SD* Standard deviation

Table 2 shows the comparison of the basic demographics of Hikikomori patients and others who visited the clinic in person at the first visit. The between-group difference of all patients was replicated in this analysis. There was no significant difference between groups in age and percentage of females. The percentage of patients with past school refusal was significantly higher in the “current” and “past” Hikikomori patients, and the number of clinics visited was significantly higher in “past” patients than “others”. “Current” (M = 12.3, SD = 4.1) and “past” (M = 10.8, SD = 4.7) Hikikomori patients were significantly more anxious than “others” (M = 8.0, SD = 4.9, *p* < .001). There was no significant difference in diagnosis between groups. The Bonferroni correction for multiple comparison setting the significance threshold as *p* < 0.008 resulted in no significant difference in the number of clinic visits among groups.

**Table 2:** *Comparison of Hikikomori patients and others (limited to “visited in person”) at a community psychiatric clinic*

	Current Hikikomori		Past Hikikomori		Others		p
	n		n		n		
Age, median (IQR)	34	39.5 (21.5-51.3)	75	33.0 (23.0-40.0)	151	30.0 (20.0-44.0)	.40
Female, n(%)	34	16 (47.1)	75	41 (54.7)	151	81 (53.6)	.75
School refusal, n(%)	29	18 (62.1)^++^	64	33 (51.6)^##^	146	24 (16.4)^++,##^	<.001
Number of Clinic visits, median (IQR)	32	1.0 (0-2.0)	69	1.0 (1.0-3.0)*	123	1.0 (0-2.0)*	.002
OASIS score, mean (SD)	30	12.3 (4.1)^#^	74	10.8 (4.7)^+^	143	8.0 (4.9)^#,+^	<.001
Diagnosis							
3F0	34	1 (2.9)	75	0 (0)	151	1 (0.7)	.056
F1		1 (2.9)		3 (4.0)		2 (1.3)	
F2		2 (5.9)		7 (9.3)		7 (4.6)	
F3		7 (20.6)		17 (22.7)		19 (12.6)	
F4		16 (47.1)		33 (44.0)		86 (57.0)	
F5		0 (0)		1 (1.3)		0 (0)	
F6		0 (0)		1 (1.3)		0 (0)	
F7		3 (8.8)		0 (0)		19 (12.6)	
F8		4 (11.8)		6 (8.0)		8 (5.3)	
F9		0 (0)		6 (8.0)		8 (5.3)	
Unclear/ Undecided		0 (0)		1 (1.3)		1 (0.7)	

*Notes.* p: ANOVA for parametric outcome and Kruskal-Wallis test for non-parametric outcome, *Adjusted Kruskal-Wallis test *p* = .001; ^#,+^ Bonferroni test *p* < .001; ^++,##^ Ryan test *p* < .001. Symbols are matched to indicate where, for the indicated two groups, the difference between those groups were significant.

*Abbreviations*: *IQR* Interquartile range, *SD* Standard deviation, *F0* Organic, including symptomatic, mental disorders, *F1* Mental and behavioral disorders due to psychoactive substance use, *F2* Schizophrenia, schizotypal and delusional disorders, *F3* Mood [affective] disorders, *F4* Neurotic, stress-related and somatoform disorders, *F5* Behavioral syndromes associated with physiological disturbances and physical factors, *F6* Disorders of adult personality and behavior, *F7* Mental retardation, *F8* Disorders of psychological development, *F9* Behavioral and emotional disorders with onset usually occurring in childhood and adolescence

We compared clinic attendance history as of the one-year follow-up between the groups (Table 3). The percentage of home visits by doctors and continuous consultation by family were significantly larger in “current” Hikikomori patients (home visit by doctor *n* = 6, 10%; continuous consultation *n* = 4, 6.7%) than “past” Hikikomori patients (*n* = 0, 0%; *n* = 0, 0%) and “others” (*n* = 0, 0%; *n* = 1, 0.6%, *p* < .001). Percentage of patients who had recovered and finalized consultation was larger in “others” (*n* = 37, 22.7%) than “current” (*n* = 4, 6.7%) and “past” hikikomori patients (*n* = 4, 4.9%, *p* < .001).

**Table 3:** The number (%) of clinic visits (attendance status) at a one-year follow-up of patients at a community psychiatric clinic

	Current Hikikomori(*n*=59)	Past Hikikomori(*n*=81)	Others(*n*=167)
Regular visit	25 (41.7)	42 (51.9)	63 (38.7)
Irregular visit	3 (5.0)	3 (3.7)	6 (3.7)
Home visit by doctor	6 (10.0)	0 (0)	0 (0)
Continuous consultation	4 (6.7)	0 (0)	1 (0.6)
Transference	7 (11.7)	9 (11.1)	14 (8.6)
Finalized consultation	4 (6.7)	4 (4.9)	37 (22.7)
Unknown	11 (18.3)	23 (28.4)	42 (25.8)

*Notes.* Chi-square test *p* < .001.

Table 4 indicates support from a nurse, PSW, OT/PT, or psychologist as of the one-year follow-up. The percentage of support from nurses was significantly higher in “current” Hikikomori patients (*n* = 9, 15%) than “others” (*n* = 6, 3.7%, *p* = .003). There was no significant difference in support from other medical staff members.

**Table 4:** The number (%) of patients being supported by clinical staff members at a community psychiatric clinic

	Current Hikikomori (*n* = 60)	Past Hikikomori (*n* = 81)	Others (*n* =163)	p
Nurse	9 (15.0)	5 (6.2)	6 (3.7)	.010
PSW	14 (23.3)	18 (22.2)	20 (12.3)	.054
OT	5 (8.3)	9 (11.1)	7 (4.3)	.13
Psychologist	3 (5.0)	7 (8.6)	17 (10.4)	.45

*Notes.* p: Chi-square test.

Table 5 indicates social function at pre- and post- follow-up. One person of the “current” Hikikomori patients in the work/school/homemaker category was a housewife. Over half of the “past” Hikikomori patients and “others” were in the work/school/homemaker category at pre- (past Hikikomori patients *n* = 43, 53.1%; others *n* = 112, 69.1%) and post- (past Hikikomori patients *n* = 45, 57.0%; others *n* = 128, 79.0%) follow-up, whereas small number of “current” Hikikomori were in the work/school/homemaker category in pre- (*n* = 1, 1.7%) and post- (*n* = 14, 23.3%) follow-up. Around half of the “current” Hikikomori patients (*n* = 31, 51.7%) were restricted in indoor activity without going out in the pre-follow-up period, which was reduced in post-follow-up (*n* = 14, 23.3%). Some “current” patients restricted their activity into their rooms prior to the the-follow-up period (*n* = 3, 5.0%).

**Table 5:** The number (%) for factors of social functioning at one-year follow-up of patients in a community psychiatric clinic

		Current Hikikomori (*n* = 60)	Past Hikikomori (*n* = 81)	Others (*n* = 162)
Work/ school/homemaker	Pre	1 (1.7)	43 (53.1)	112 (69.1)
	Post	14 (23.3)	45 (57.0)	128 (79.0)
Day/night care	Pre	0 (0)	0 (0)	0 (0)
	Post	6 (10.0)	4 (5.1)	7 (4.3)
Going out freely	Pre	4 (6.7)	24 (29.6)	37 (22.8)
	Post	3 (5.0)	21 (26.6)	13 (8.0)
Going out with restriction	Pre	19 (31.7)	12 (14.8)	11 (6.8)
	Post	14 (23.3)	9 (11.4)	12 (7.4)
Indoor activity without going out	Pre	31 (51.7)	2 (2.5)	2 (1.2)
	Post	19 (31.7)	0 (0)	2 (1.2)
Activity restricted to their room	Pre	3 (5.0)	0 (0)	0 (0)
	Post	4 (6.7)	0 (0)	0 (0)
Unknown	Pre	2 (3.3)	0 (0)	0 (0)
	Post	0 (0)	0 (0)	0 (0)

We analyzed factors contributing to clinic attendance and social function, which were the primary outcomes. Table 6 shows the results of uni- and multivariable logistic regression for the clinic attendance variable. The results of the multivariable logistic regression indicated that “current” Hikikomori status at baseline predicted significantly less regular attendance/completed treatment at the clinic (odds ratio [OR] 0.43, 95% confidence interval [CI] 0.22–0.83, *p* = .012), and the support from PSW and OT/PT significantly predicted more regular attendance/completed treatment (PSW OR = 2.35, 95% CI 1.14–4.86, *p* = .021; OT OR = 6.07, 95% CI 1.28–28.71, *p* = .023). Being female, and having support from a nurse, PSW, or OT/PT significantly related to maintained contact with patients at the clinic or at home (female OR 1.66, 95% CI 1.01–2.73, *p* = .046; support from nurse OR = 8.44, 95% CI 1.05–68.11, *p* = .045; PSW OR 2.44, 95% CI 1.12–5.32, *p* < .025; OT/PT OR 10.06, 95% CI 1.25–80.89, *p* = .030).

**Table 6:** The logistic regression analysis on clinic attendance status with dependent variable “finalized consultation/ regular visit” and dependent variable “finalized consultation/regular visit/irregular visits/home visits by a doctor”

	Univariable OR			Multivariable OR		
†	OR	95%CI	p	OR	95%CI	p
Age	1.01	1.00 to 1.03	.18	1.01	0.99 to 1.03	.34
Female	1.45	0.92 to 2.28	.11	1.44	0.89 to 2.34	.14
Current Hikikomori	0.59	0.33 to 1.07	.08	0.43	0.22 to 0.83	.012
Past Hikikomori	0.83	0.48 to 1.42	.49	0.66	0.37 to 1.18	.16
Other (reference)	1			1		
Nurse	4.80	1.38 to 16.7	.013	3.74	0.95 to 14.68	.059
PSW	2.63	1.34 to 5.15	.005	2.35	1.14 to 4.86	.021
OT/PT	8.17	1.87 to 35.6	.005	6.07	1.28 to 28.71	.023
Psychologist	2.24	0.92 to 5.47	.076	1.74	0.66 to 4.59	.26
	Univariable OR			Multivariable OR		
‡	OR	95%CI	p	OR	95%CI	p
Age	1.01	0.99 to 1.03	.20	1.01	0.99 to 1.03	.43
Female	1.64	1.03 to 2.61	.039	1.66	1.01 to 2.73	.046
Current Hikikomori	0.93	0.50 to 1.72	.81	0.70	0.36 to 1.37	.30
Past Hikikomori	0.82	0.48 to 1.43	.49	0.65	0.36 to 1.17	.15
Other (reference)	1			1		
Nurse	12.73	1.68 to 96.16	.014	8.44	1.05 to 68.11	.045
PSW	2.90	1.39 to 6.02	.004	2.44	1.12 to 5.32	.025
OT	13.44	1.78 to 101.32	.012	10.06	1.25 to 80.89	.030
Psychologist	2.73	1.00 to 7.43	.049	2.31	0.80 to 6.68	.12

*Notes.* Multivariate analysis included all the variables in the table. †Dependent variable: finalized consultation/ regular visit.

*Notes.* Multivariate analysis included all the variables in the table. ‡ Dependent variable: finalized consultation/regular visit/irregular visits/home visits by a doctor. *Abbreviations*: *PSW* Psychiatric social worker, *OT/PT* Occupational therapist/physical therapist.

*Abbreviations*: *PSW* Psychiatric social worker, *OT/PT* Occupational therapist/physical therapist.

Table 7 shows the results of uni- and multivariable logistic regression for social function. The result of multivariable logistic regression showed that “current” status and “past” Hikikomori experience at baseline significantly decreased and “female” significantly increased the work/school status at the one-year follow-up (current Hikikomori OR = 0.30, 95% CI 0.13–0.68, *p* = .004; past Hikikomori OR = 0.40, 95% CI 0.20–0.82, *p* = .012; female OR = 1.96, 95% CI 1.04–3.70, *p* = .037).

**Table 7:** The logistic regression analysis on social functioning with the dependent variable “work/school” and the dependent variable “work/school/day-night care”

	Univariable OR			Multivariable OR		
†	OR	95%CI	p	OR	95%CI	p
Age	1.01	0.99 to 1.04	.21	1.02	0.99 to 1.04	.17
Female	2.24	1.23 to 4.07	.008	1.96	1.04 to 3.70	.037
Current Hikikomori	0.27	0.12 to 0.59	.001	0.30	0.13 to 0.68	.004
Past Hikikomori	0.39	0.20 to 0.78	.007	0.40	0.20 to 0.82	.012
Other (reference)	1			1		
Nurse	0.50	0.17 to 1.54	.23	0.58	0.17 to 2.02	.39
PSW	0.70	0.34 to 1.46	.35	0.85	0.39 to 1.89	.69
OT	0.32	0.10 to 1.02	.053	0.36	0.09 to 1.40	.14
Psychologist	1.50	0.56 to 3.98	.42	1.78	0.57 to 5.59	.32
	Univariable OR			Multivariable OR		
‡	OR	95%CI	p	OR	95%CI	p
Age	1.01	0.99 to 1.03	.36	1.01	0.98 to 1.03	.49
Female	1.58	0.88 to 2.82	.12	1.56	0.82 to 2.96	.18
Current Hikikomori	0.32	0.15 to 0.67	.003	0.30	0.13 to 0.67	.003
Past Hikikomori	0.34	0.17 to 0.69	.003	0.28	0.14 to 0.60	.001
Other (reference)	1					
Nurse	3.23	1.14 to 9.18	.028	3.28	0.95 to 11.30	.06
PSW	1.68	0.81 to 3.48	.16	1.29	0.57 to 2.91	.54
OT	5.05	1.55 to 16.46	.007	4.49	1.16 to 17.43	.030
Psychologist	2.79	0.98 to 7.92	.054	1.57	0.47 to 5.26	.47

*Notes.* Univariate analysis was adjusted for baseline social functioning, Multivariate analysis included all the variables in the table. †Dependent variable: work/school.

*Notes*. Univariate analysis was adjusted for baseline social functioning, Multivariate analysis included all the variables in the table. ‡Dependent variable: work/school/day-night care.

*Abbreviations*: *PSW* Psychiatric social worker, *OT/PT* Occupational therapist/physical therapist.

The Bonferroni correction for multiple comparison setting the significance threshold as *p*<0.025 for the two primary outcomes did not change the significance according to the Hikikomori status.

We conducted the same analysis with the “current” Hikikomori patients. The attendance in person at the first visit was included as an independent variable this time. Factors that significantly predicted the regular visit/completed treatment by the logistic regression were as follows: the attendance in person at the first visit (univariable OR = 6.97, 95% CI 2.18–22.26, *p* = .001; multivariable OR = 21.59, 95% CI 3.1– 150.30, *p*=.002), support by nurse (univariate OR=11.43, 95%CI 1.33–98.34, *p*=.027; multivariable OR=10.00, 95% CI 0.73–137.51, *p* = .085), and support by PSW (univariate OR = 3.55, 95% CI 0.97–13.03, *p* = .056; multivariable OR = 15.24, 95% CI 1.62–143.26, *p* = .017). The percentage of patients who had attended the clinic regularly as of their one-year follow-up was 79.3% (*n* = 23) for those who had attended in person at first visit and 35.5% (*n* = 11) for “others.”

Factors that significantly predicted the maintained contact with patients at clinic or home were as follows: attendance in person at the first visit (univariable OR=3.79, 95%CI 1.26 to 11.46, *p*=.018; multivariable OR=4.74, 95%CI 1.04 to 21.58, *p*=.044) and “support by PSW” (univariable OR=4.62, 95%CI 0.93 to 23.01, *p*=.062; multivariable OR=14.43, 95%CI 1.29 to 161.42, *p*=.03).

The “female” factor significantly increased patients’ work/school/homemaker status at a one-year follow-up (univariable OR = 4.16; 95% CI 1.06–19.50; *p* = .041, adjusted for baseline social function; multivariable OR=6.23, 95%CI 0.95 to 40.83, *p*=.057). No other factors significantly predicted the work/school/homemaker social function. No factors predicted work/school status using the multivariable logistic regression.

## Discussion

This is the first study to clarify the percentage of first-visit “current” and “past” Hikikomori patients in a community private clinic and compare their outcomes at one-year follow-up. The results indicated that 19.7% and 26.6% of first-visit patients were “current” and “past” Hikikomori patients, respectively. That is, about half of the first-visit patients are currently experiencing or have experienced Hikikomori. However, the one-year outcomes of “current” Hikikomori patients were poor, as shown in regular attendance/completed treatment in clinic and work/school status. Among the current Hikikomori patients, the attendance in person at the first visit and support from nurse or PSW increased regular attendance/completed treatment, whereas no factors related to increased work/school status except for female gender.

The aging of patients with Hikikomori has been attracting attention in Japan since around 2014, when a book *Adult Hikikomori* (*Otona no Hikikomori*) was published that pointed out this serious situation [5]. In addition to the aging of Hikikomori patients, the aging of their parents is an accompanied problem. This is called the “8050” problem, meaning 80-year-old parents are supporting 50-year-old Hikikomori patients. Both the parents and the patients worry about the future after the death of the parents. In fact, the survey in 2018 reported that the number of Hikikomori between the ages of 40 and 64 is estimated at about 613,000 for the country of Japan [4]. The results of previous studies implied that the mean age of Hikikomori patients in psychiatric institutions was 19.0 in 2007, and the age of those in the community clinics was 28.9 in 2010 [12, 23].

The present study added new insights regarding the distribution of age of patients with Hikikomori. The age of current Hikikomori patients in our study was bimodal in distribution, peaking at 20, 40, and 45. This means that the aging of Hikikomori patients is real but that new, young Hikikomori patients are still appearing. The smaller frequency of Hikikomori patients aged between 25 and 35 was an “blank period” around the ages of those visiting clinics. Supposing that the parents of these patients were amongst the working population, the pressure on Hikikomori patients from others or by themselves may be weaker than other age groups. However, patients may be urged to get jobs around the age of 20 as well as at 40 when parents are facing their retirement, relating to the occurrence of clinic visits for patients of those ages. The factors promoting patients and parents to visit clinics should be investigated.

Some studies on the prevalence of Hikikomori and one on student apathy and withdrawal in Japan reported that Hikikomori was more prevalent among males than among females [2–4, 12, 23, 24], whereas a study on Hikikomori prevalence in Hong-Kong and home visitation program in Korea showed no significant difference in gender [14, 25]. We found no difference in gender among “current,” “past” Hikikomori, and “other” patients. If Hikikomori is prevalent in males, they may be less motivated or find it more difficult to seek clinic consultation. However, considering the studies in Hong-Kong, Korea, and the present study, we should be cautious about assuming it is more common in males.

The demographic comparison speaks to the causes and progression of Hikikomori state. “Current” and “past” Hikikomori patients experienced more school refusal and felt more anxious than other patients. One of the main pathologies of Hikikomori may be anxiety, leading to school refusal and present anxiety. Alternatively, school refusal may promote anxiety, leading to Hikikomori. Patients with Hikikomori may tend to visit more clinics than other patients. This may reflect Hikikomori patients’ anxiety or mean that no appropriate treatment for Hikikomori patients was provided. Patients with Hikikomori may be continuing to seek help more than other patients.

All the present and past Hikikomori patients had psychiatric disorders. The original definition of Hikikomori does not include people with psychiatric disorders. However, the newest definition by the Japanese Ministry of Health, Labor, and Welfare signifies inclusion of patients with psychiatric disorders. The present study supports the validity of the latter definition. Although it was not significant, the percentage of mood disorders was relatively large among current and past Hikikomori patients than other patients, which should be confirmed by studies with larger sample sizes. Identifying a pathological relationship between Hikikomori and mood disorders may be one of the research implications.

Of optimistic note was the finding that about 40% of “current” Hikikomori patients regularly visited clinics at their one year follow up, although the “current” Hikikomori state significantly lowered the visit’s likelihood compared to “other” patients. The factors promoting regular visits were attendance in person at first visit, support from nurses, and support from PSW. Interventions encouraging Hikikomori patients to visit clinics in person are needed, and interventions directed at parents may also be effective. The Japanese Ministry of Health, Labor, and Welfare conducted research on the living conditions of citizens aged between 40 and 64 in 2018. The percentage of Hikikomori patients who lived alone was only 10.6% and the number of those who lived with their mother or father was 53.2% and 25.5%, respectively. The efficacy of parental intervention has been shown for supporting parenting for anxious childhood emotions [13]. Parental involvement in treatment is efficacious to reducing youth anxiety [17]. Additionally, the present study may support the importance of case management by clinical team members, especially nurses and PSW. A meta-analysis indicated intensive case management for severe mental illness reduced hospitalization [1]. The results may apply to Hikikomori patients to promote their clinical attendance. However, patients with good prognoses could have been better supported by clinical staff members in the present study, possibly creating an overestimate of the results.

Unfortunately, no factor except for female gender promoted work/school status at one-year follow-up. There is an apparent gap between regular visits and social function. This may indicate that more years of follow-up are needed to be able to see improvements in social function.

The present study has several limitations. First, the setting is a relatively large community clinic with 36 clinical workers, including part-time workers, and it somewhat focuses on supporting patients with Hikikomori. Therefore, these results may be more optimistic than representative of general community clinics. The generalizability of the present results therefore cannot be assumed. Second, the sample size was small, which may have caused an underestimation of the influence of the variables on outcomes. The number of case categories was sometimes very small. They include contact with different kinds of healthcare providers and diagnoses of psychiatric disorders. The present study cannot make generalizable conclusions due to these points. Therefore, studies with larger sample sizes should be conducted. Third, the Hikikomori state and school refusal were evaluated by subjective questionnaires. The agreement of answers from both patients and their families should be tested for more objectivity. However, there was no essential difference between the analyses using data from all subjects and data confined to subjects who attend the clinic in person. This indicates that the influence of bias according to the responder was negligible, or non-existent. Fourth, the follow-up period was short, as it may take longer than a year for improvements in social functioning, and the present results regarding social functioning may underestimate the effect of factors. Fifth, data about participants’ living circumstances were not collected, which may be a confounding variable.

## Conclusions

The percentage of patients who experience Hikikomori could be large. The “current” Hikikomori state may lower patients' clinic attendance and their social function at a one-year follow-up. Hikikomori appears to be more difficult to successfully treat than many other psychiatric disorders. That fact, combined with the social situation of aging parents and patients, suggests further research into successful treatments, interventions for parents, and examination of the causes of Hikikomori should be considered a priority.

## Data Availability

The dataset supporting the conclusion of the current study is available from the corresponding author on reasonable request.

## Abbreviations

OASIS: The Overall Anxiety Severity and Impairment Scale
PSW: Psychiatric social worker
OT/PT: Occupational/physical therapist
SD: Standard deviation
OR: Odds ratio
CI: Confidence interval
IQR: Interquartile range
SPACE: Supporting parenting for anxious childhood emotions
